# Short–term effects of neoadjuvant chemoradiation therapy on anorectal function in rectal cancer patients: a pilot study

**DOI:** 10.1186/1748-717X-8-203

**Published:** 2013-08-20

**Authors:** Bong-Hyeon Kye, Hyung-Jin Kim, Jun-Gi Kim, Sung-Hwan Kim, Byoung-Yong Shim, Nan-Sook Lee, Hyeon-Min Cho

**Affiliations:** 1 Department of Surgery, St. Vincent Hospital, The Catholic University of Korea, Suwon, Korea; 2 Department of Surgery, Seoul St. Mary’s Hospital, The Catholic University of Korea, Seoul, Korea; 3 Department of Radiation Oncology, St. Vincent Hospital, The Catholic University of Korea, Suwon, Korea; 4 Department of Medical Oncology, St. Vincent Hospital, The Catholic University of Korea, Suwon, Korea

**Keywords:** Anorectal function, Neoadjuvant chemoradiation, Manometry, Rectal cancer

## Abstract

**Background:**

Neoadjuvant chemoradiation therapy followed by curative surgery has gained acceptance as the therapy of choice in locally advanced rectal cancer. However, deterioration of anorectal function after long-course neoadjuvant chemoradiation therapy combined with surgery for rectal cancer is poorly defined. The aim of this study was to evaluate the physiological and clinical change of anorectal function after neoadjuvant chemoradiation therapy for rectal cancer.

**Methods:**

We analyzed 30 patients on whom preoperative anorectal manometry data were available both before and after chemoradiation from October 2010 to September 2011. All patients underwent long-course neoadjuvant chemoradiation therapy. We compared manometric parameters between before and after neoadjuvant chemoradiation therapy.

**Results:**

Of 30 patients, 20 were males and 10 females. The mean age was 64.9 ± 9.9 years (range, 48-82). Before nCRT, the rectal compliance was higher in patients with ulceroinfiltrative type (P = 0.035) and greater involvement of luminal circumference (P = 0.017). However, there was the tendency of increased rectal sensory threshold for desire to defecate when the patient had decreased circumferential ratio of the tumor (P = 0.099), down-graded T stage (P = 0.016), or reduced tumor volume (P = 0.063) after neoadjuvant chemoradiation.

**Conclusions:**

Neoadjuvant chemoradiation therapy did not significantly impair overall sphincter function before radical operation. The relationship between tumor response of chemoradiation and sensory threshold for desire to defecate may suggest that neoadjuvant chemoradiation may be helpful for defecatory function as well as local disease control, at least in the short-term period after the radiation in locally advanced rectal cancer patients.

## Background

Neoadjuvant chemoradiation therapy (nCRT) followed by curative surgery has become the therapy of choice in locally advanced rectal cancer. The nCRT was introduced to improve local control and some studies have demonstrated a significant benefit from this treatment with reduced local recurrence rates [1]. Low anterior resection (LAR) with total mesorectal excision (TME) is the standard surgical procedure in sphincter-preserving surgery for rectal cancer [2]. Currently, a combined-modality therapy including surgery, radiotherapy, and chemotherapy is recommended for the majority of patients with stage II or III rectal cancer.

However, deterioration of anorectal function after nCRT combined with sphincter-preserving surgery (SPS) for rectal cancer may happen. Although factors that may affect on postoperative bowel function are not predictable and not well understood, nCRT and LAR with TME can be important ones that may influence anorectal function [3-5]. Many investigators have reported defecatory dysfunction such as frequent defecation, urgency, and incontinence after sphincter-saving procedure, especially with low colorectal or coloanal anastomosis [6,7]. Worsening of anorectal function after nCRT for locally advanced rectal cancer is often referred as radiation damage of the anorectum and pelvic floor [8,9].

Until recently, most of the studies about the effects of nCRT on anorectal function have assessed pre-nCRT and long-term postoperative results [3-6,8]. However, SPS alone could also adversely affect the outcome of anorectal function and it could introduce a significant bias in the assessment of the impact of nCRT. There are a few studies that reported short-term effect of nCRT on anorectal function [10,11]. In that studies, the authors found no significant changes of manometric data after nCRT and concluded that fecal incontinence after SPS should not be the result of radiotherapy. However, they did not evaluate the relationship between the tumor response and the change of anorectal function.

In this study, we aimed to evaluate the preoperative short-term effects of nCRT on anorectal function and the relationship between the tumor response for nCRT and the change of anorectal function.

## Methods

We prospectively collected manometric data before and after nCRT in patients who were diagnosed with adenocarcinoma of rectum below 12 cm from the anal verge by rigid proctosigmoidoscopy before nCRT from October 2010. Among these patients, 30 patients whose preoperative manomertic data were available at both before and after nCRT from October 2010 to September 2011 were enrolled in this study. After obtaining review board approval from our institute (VC12RISI0016), we analyzed manometric data and clinical information of these 30 patients retrospectively.

Clinical stage was determined according to the American Joint Committee on Cancer Six Staging System. All 30 patients had clinical T3 to T4 or N positive tumors. All of the analyzed patients received neoadjuvant radiotherapy with conventional fractionation as follows: 1.8 Gy per day; five fractions per week; and total dose, 50.4 Gy/28 fractions (45 Gy/25 fractions initially to the whole pelvis, followed by 5.4 Gy/3 fractions as a boost to the gross tumor). All of the patients received two cycles of concurrent chemotherapy with radiotherapy (5-fluorouracil [5-FU], 400 mg/m^2^ [IV] 1 h before radiotherapy and leucovorin, 20 mg/m^2^ [IV] immediately before each dose of 5-FU on days 1–5 and days 29–33).

Anorectal manometry was performed before nCRT and three to four weeks after the end of nCRT with the water-perfusion technique using an 8-channel Micro Tip catheter connected to a perfusion pump (POLYGRAF ID; Alpine Biomed, Denmark). Manometric data including mean resting pressure (MRP), maximum squeezing pressure (MSP), the percentages of asymmetry of resting (R asymmetry) and squeezing (S asymmetry) sphincter, the length of high-pressure zone (HPZ) at resting and squeezing, recto-anal inhibitory reflex (RAIR), rectal sensory threshold (RST), and rectal compliance. RST was measured via determination of threshold volume of the first minimal sensation, desire to defecate, and maximal tolerance. All manometry procedures were performed by a single practitioner who has performed anal physiologic test over 5 years.

We compared manometric data before nCRT according to tumor location from the anal verge, tumor gross morphology, tumor circumferential ratio (CIR) and T stage. Also, we evaluated tumor response after nCRT with above factors adding tumor volume. We divided our patients into two groups according to tumor location from the anal verge whether the lower margin of tumor was >5 cm or ≤ 5 cm from anal verge by rigid proctosigmoidoscopy. For patients with tumor below 5 cm from the anal verge, the field of additional booster on peritumoral region included the anal canal. However, in tumor above 5 cm from the anal verge, the anal canal and perineum were excluded from the field of additional booster. This was the reason that we divided out patients with 5 cm criteria. Tumor gross morphology on flexible colonofiberscopy was classified to three categories; fungating, ulcerofungating, and ulceroinfiltrative. Tumor CIR was defined as a ratio of the circumference of infiltrative component of tumor to luminal circumference. We used trans-rectal ultrasound to measure tumor CIR. CIR was classified into four categories; CIR ≤ 1/4, CIR ≤ 1/2, CIR ≤ 3/4, and CIR >3/4. T stage was also measured by trans-rectal ultrasound. We defined reduction of tumor volume as more than 70% of decreased tumor volume after nCRT by CT volumetry. Rigid proctosigmoidoscopy, flexible colonofiberscopy, and trans-rectal ultrasound were performed by the colorectal surgeons whose sub-specialty is colorectal disease in Korea and are co-authors in this study.

The difference of manometric data between before and after nCRT was measured by subtraction from manometric data before nCRT to that after nCRT. The negative value means that value of manometric data after nCRT was increased comparing to that before nCRT.

Continuous variables between two groups were compared using the Student’s t-test and expressed as mean ± SD. Categorical variables were analyzed with χ^2^ test. Although significance was defined as P ≤ 0.05, P ≤ 0.1 was regarded as marginal significance. All statistical analyses were performed using the Statistical Package of the Social Sciences (SPSS) version 15.0 for Windows (SPSS, Inc, Chicago, IL).

## Results

Of 30 patients, there were 20 males and 10 females. The mean age was 64.9 ± 9.9 years (range, 48–82). All patients did not experience any radiation-related or chemotherapy-related major adverse effect, more than grade 3 reaction according to the Common Toxicity Criteria. There was a marginal difference in the maximal rectal sensory threshold between before and after nCRT (179.3 ± 63.7 ml vs 151.7 ± 53.4 ml, P = 0.073) without a difference in rectal compliance (P = 0.638) (Table 1). There were four patients whose RAIR were absent before CRT. However, this reflex was recovered after nCRT in these four patients.

**Table 1:** Comparison of manometric data between before and after nCRT in 30 patients

	**Before nCRT (N = 30)**	**After nCRT (N = 30)**	**P-value**
MRP (mmHg)	93.5 ± 44.7	80.3 ± 32.4	0.195
R asymmetry (%)	22.3 ± 12.9	23.9 ± 9.7	0.572
MSP (mmHg)	348.8 ± 177.2	285.2 ± 111.7	0.102
S asymmetry (%)	17.2 ± 11.6	16.3 ± 4.8	0.667
HPZ length at rest (cm)	1.8 ± 0.6	1.8 ± 0.8	0.789
HPZ length at squeezing (cm)	2.2 ± 0.5	2.3 ± 0.8	0.634
RAIR (ml)	37.3 ± 13.6	40.7 ± 9.8	0.281
Rectal sensory threshold (ml)			
First	63.0 ± 32.1	57.3 ± 18.9	0.408
Desire to defecate	117.3 ± 46.5	108.7 ± 42.3	0.453
Maximal	179.3 ± 63.7	151.7 ± 53.4	0.073
Rectal compliance (ml/mmHg)	1.4 ± 0.8	1.3 ± 0.7	0.638

*MRP* Mean Resting Pressure, *R asymmetry* asymmetry of the resting sphincter, *MSP* Maximal Squeezing Pressure, *S asymmetry* asymmetry of the squeezing sphincter, *HPZ* High Pressure Zone, *RAIR* Recto-Anal Inhibitory Reflex, *nCRT* neoadjuvant chemoradiation therapy.

In manometry performed before nCRT, the maximal RST was marginally higher in patients with ulceroinfiltrative type than ulcerofungating type (212.5±80.3 ml vs 167.2±53.7ml, P = 0.086) and the rectal compliance was significantly higher in patients with ulceroinfiltrative type than ulcerofungating type (1.9 ± 1.1 ml/mmHg vs 1.2 ± 0.6 ml/mmHg, P = 0.035). Also, as smaller involvement of luminal circumference, the maximal RST was higher marginally (P = 0.083) and the rectal compliance was significantly higher (P = 0.017) (Table 2).

**Table 2:** Comparison of manometric data before nCRT according to each tumor characteristics

	**Tumor location**			**Gross morphology**			**CIR**				**T stage**		
	**>5 cm (n = 18)**	**≤5 cm (n = 12)**	**P-value**	**UF (n = 22)**	**UI (n = 8)**	**P-value**	**R1 (n = 15)**	**R2 (n = 10)**	**R3 (n = 5)**	**P-value**	**T3 (n = 27)**	**T4 (n = 3)**	**P-value**
MRP (mmHg)	89.4 ± 41.7	99.6 ± 50.1	0.550	97.8 ± 46.4	81.7 ± 39.9	0.393	79.6 ± 38.7	99.3 ± 38.4	123.5 ± 62.9	0.144	88.4 ± 43.6	138.8 ± 26.6	0.063
R asymmetry (%)	20.7 ± 5.8	24.6 ± 19.5	0.427	22.8 ± 14.5	20.8 ± 7.5	0.725	21.4 ± 6.8	17.6 ± 5.8	34.1 ± 27.3	0.059	23.1 ± 13.3	14.9 ± 3.9	0.310
MSP (mmHg)	313.7 ± 116.5	401.3 ± 238.2	0.189	345.9 ± 200.8	356.5 ± 94.4	0.888	319.7 ± 91.5	353.6 ± 121.6	425.9 ± 392.7	0.523	351.1 ± 183.7	328.5 ± 124.5	0.839
S asymmetry (%)	15.9 ± 4.1	19.2 ± 17.9	0.470	18.2 ± 13.3	14.7 ± 3.2	0.485	15.3 ± 4.2	13.9 ± 3.3	29.4 ± 25.6	0.029	17.8 ± 12.1	12.1 ± 2.2	0.424
HPZ length at rest (cm)	1.7 ± 0.6	1.9 ± 0.5	0.512	1.8 ± 0.6	1.6 ± 0.4	0.438	1.6 ± 0.5	1.7 ± 0.6	2.1 ± 0.7	0.416	1.7 ± 0.6	1.8 ± 0.7	0.829
HPZ length at squeezing (cm)	2.3 ± 0.5	2.1 ± 0.3	0.147	2.2 ± 0.5	2.1 ± 0.3	0.251	2.0 ± 0.3	2.3 ± 0.6	2.6 ± 0.4	0.059	2.1 ± 0.4	2.8 ± 0.7	0.017
RAIR (ml)	36.7 ± 14.1	38.3 ± 13.4	0.749	38.2 ± 8.5	35.0 ± 23.3	0.581	40.0 ± 13.1	36.0 ± 12.6	32 ± 17.8	0.504	37.1 ± 14.3	40.0 ± 0.1	0.728
Rectal sensory threshold (ml)													
First	65.6 ± 37.9	59.2 ± 21.5	0.602	63.2 ± 35.1	62.5 ± 23.7	0.960	71.3 ± 41.5	59.0 ± 17.9	46.0 ± 0.5	0.258	62.9 ± 32.6	63.3 ± 32.1	0.985
Desire to defecate	116.1 ± 45.8	119.2 ± 49.4	0.863	116.8 ± 43.4	118.7 ± 57.4	0.922	204.0 ± 66.2	162.0 ± 57.3	179.3 ± 63.7	0.297	117.1 ± 45.1	120.0 ± 69.2	0.919
Maximal	174.4 ± 57.2	186.7 ± 74.5	0.615	167.2 ± 53.7	212.5 ± 80.3	0.086	204.0 ± 66.2	162.0 ± 57.3	140.0 ± 41.8	0.083	177.1 ± 65.4	200.0 ± 50.0	0.563
Rectal compliance (ml/mmHg)	1.4 ± 0.8	1.3 ± 0.7	0.852	1.2 ± 0.6	1.9 ± 1.1	0.035	1.8 ± 0.9	1.1 ± 0.5	0.8 ± 0.5	0.017	1.4 ± 0.8	1.0 ± 0.2	0.346

*MRP* Mean Resting Pressure, *R asymmetry* asymmetry of the resting sphincter, *MSP* Maximal Squeezing Pressure, *S asymmetry* asymmetry of the squeezing sphincter, *HPZ* High Pressure Zone, *RAIR* Recto-Anal Inhibitory Reflex, *UF* Ulcerofungating type, *UI* Ulceroinfiltrative type, *CIR* Circumferential ratio, *R1* 1/4 < CIR ≤ 2/4, *R2* 2/4 < CIR ≤ 3/4, *R3* CIR > 3/4.

When we considered the effect of nCRT on tumor, there were twenty two patients who underwent the change of tumor gross morphology after nCRT, ten patients with reduced CIR after nCRT, eight patients with down-staging in T-stage, and four patients with more than 70% of volume reduction of tumor after nCRT. When we considered the effect of nCRT on anorectal function, there were no significant differences on the changes of manometric data between before and after nCRT according to the tumor location from the anal verge and the change of tumor gross morphology. Patients who had tumor with reduced CIR after nCRT had the tendency of improved RST for ‘desire to defecate’ (P = 0.099). Those patients with down-staging in T stage showed elongated HPZ length (P = 0.030), decreased volume for RAIR (P = 0.029), and improved RST for ‘desire to defecate’ (P = 0.016). Although there was no significance, patients with tumor volume reduction after nCRT showed the tendency of increased RST for ‘desire to defecate’ (P = 0.063) (Table 3).

**Table 3:** Comparison of the difference of manometric data between before and after nCRT according to each tumor characteristics

	**Tumor location**^**†**^			**Gross morphology**			**CIR**			**T stage**			**Tumor volume**		
	**>5 cm (n = 18)**	**≤5 cm (n = 12)**	**P-value**	**No change (n = 8)**	**Change (n = 22)**	**P-value**	**No reduced (n = 20)**	**Reduced (n = 10)**	**P-value**	**No reduced (n = 22)**	**Reduced (n = 8)**	**P-value**	**No reduced (n = 26)**	**Reduced (n = 4)**	**P-value**
MRP (mmHg)	8.2 ± 26.9	20.7 ± 39.2	0.304	12.1 ± 24.5	13.6 ± 35.3	0.914	9.1 ± 25.4	21.5 ± 43.6	0.331	16.2 ± 32.6	4.9 ± 32.3	0.404	12.4 ± 33.8	18.6 ± 23.5	0.726
R asymmetry (%)	−3.8 ± 5.9	1.5 ± 23.7	0.371	−3.5 ± 5.0	−1.1 ± 17.9	0.700	−3.7 ± 5.7	2.5 ± 26.0	0.311	−10.1 ± 15.9	−3.4 ± 15.5	0.731	−2.2 ± 16.6	1.5 ± 3.1	0.670
MSP (mmHg)	39.5 ± 73.4	99.6 ± 261.9	0.362	21.9 ± 73.4	78.6 ± 197.1	0.438	35.2 ± 71.8	120.1 ± 284.1	0.212	74.6 ± 196.5	33.1 ±85.8	0.571	63.2 ± 184.2	65.5 ± 89.9	0.981
S asymmetry (%)	−0.7 ± 4.3	3.6 ±18.5	0.348	0.3 ± 3.8	1.3 ± 13.9	0.843	−1.2 ± 4.5	5.4 ± 19.8	0.162	1.2 ± 13.9	0.4 ±3.6	0.869	0.8 ± 12.9	2.0 ±1.2	0.860
HPZ length at rest (cm)	−0.1 ± 0.6	0.08 ±0.7	0.345	0.2 ± 0.6	−0.1 ± 0.6	0.140	−0.08 ± 0.6	0.02 ± 0.7	0.677	0.1 ± 0.5	−0.4 ±0.6	0.030	0.008 ±0.5	−0.4 ±0.8	0.215
HPZ length at squeezing (cm)	−0.02 ± 0.8	−0.2 ± 0.8	0.612	0.01 ± 1.0	−0.1 ± 0.7	0.696	−0.1 ± 0.9	−0.02 ± 0.4	0.762	−0.1 ± 0.8	0.06 ± 0.7	0.551	−0.06 ± 0.6	−0.3 ±0.3	0.658
RAIR (ml)	−2.2 ± 18.1	−5.0 ± 15.1	0.663	−7.5 ± 18.3	−1.8 ± 16.2	0.419	−4.0 ± 17.9	−2.0 ± 14.7	0.763	−7.3 ± 15.8	7.5 ± 14.8	0.029	−4.6 ± 17.3	5.0 ±10.0	0.291
Rectal sensory threshold (ml)															
First	8.3 ± 38.8	1.7 ±21.2	0.593	16.25 ± 56.6	1.8 ± 18.7	0.292	4.0 ± 10.5	9.0 ± 56.5	0.700	10.0 ± 32.9	−6.2 ± 30.7	0.235	7.3 ±31.3	−5.0 ±44.3	0.493
Desire to defecate	9.4 ± 51.5	7.5 ± 51.9	0.920	31.3 ± 64.2	0.5 ± 43.8	0.145	19.5 ± 32.4	−13.0 ± 72.9	0.099	21.8 ± 41.7	−27.5 ± 58.5	0.016	15.4 ±45.3	−35.0 ± 70.0	0.063
Maximal	28.9 ± 55.2	25.8 ±49.4	0.878	41.3 ± 57.2	22.7 ± 50.6	0.399	36.5 ± 50.1	10.0 ± 54.2	0.194	30.9 ±58.1	18.8 ± 31.8	0.581	29.6 ± 54.9	15.0 ±30.0	0.610
Rectal compliance (ml/mmHg)	0.2 ± 0.7	−0.07 ± 0.5	0.283	0.1 ± 0.7	0.07 ± 0.7	0.794	0.2 ± 0.7	−0.1 ± 0.5	0.206	0.1 ±0.7	0.006 ± 0.5	0.675	0.1 ± 0.7	−0.3 ± 0.5	0.223

*MRP* Mean Resting Pressure, *R asymmetry* asymmetry of the resting sphincter, *MSP* Maximal Squeezing Pressure, *S asymmetry* asymmetry of the squeezing sphincter, *HPZ* High Pressure Zone, *RAIR* Recto-Anal Inhibitory Reflex, *CIR* Circumferential ratio.

† For patients with tumor below 5 cm from the anal verge, the field of additional booster on peritumoral region included the anal canal. However, in tumor above 5 cm from the anal verge, the anal canal and perineum were excluded from the field of additional booster.

* The negative value means that value of manometric data after nCRT was increased than that before nCRT.

## Discussion

The majority of patients with rectal cancer have received external pelvic radiation therapy for local control of tumors. The nCRT has been preferred due to lower 5-year locoregional recurrence and better sphincter preservation [1]. Anorectal function may deteriorate after SPS with TME. Some studies have described the “anterior resection syndrome” as an increased number of daily bowel movements, clustering, anal incontinence, and soiling after SPS [12]. An increase in number of stools, frequent use of pads, urgency of defecation, impaired sensory perception, and fecal incontinence may occur after pelvic radiation [9,13-15]. Hence, it is well conceivable that nCRT may cause additional damage to sphincter function after SPS with TME. However, the data about short-term preoperative change of anorectal function based on manometric data after nCRT were limitd [10,11]. Especially, the report for the change of manometric data regarding to tumor response after nCRT has not been published.

In this study, we examined the effect of nCRT on anorectal function in three aspects; the change of anorectal function by rectal irradiation, that according to whether anal sphincter was irradiated or not by additional booster on peritumoral region, and that according to the tumor response.

Firstly, we investigated the change of anorectal function by rectal irradiation. Some studies about postoperative change of anorectal function demonstrated that the RAIR is stimulated by intramural nervous pathways. These nervous pathways might be injured during the dissection of the rectum, then the RAIR will be absent postoperatively [16,17]. However, two studies about the short-term preoperative change of anorectal function after nCRT demonstrated that the RAIR is present in all patients both before and after nCRT and there is no change in RAIR between them [10,11]. In our study, the RAIR was not changed after nCRT (37.3 ± 13.6 mL vs 40.7 ± 9.8 mL, P = 0.281). There were four patients who were absent of the RAIR before nCRT, however, the RAIR was present after nCRT in these four patients. This finding may imply that the reflex mechanism was not damaged by nCRT in short-term period after nCRT. When we analyzed the data from all 30 patients, the maximal RST was decreased after nCRT (179.3 ± 63.7 mL vs 151.7 ± 53.4 mL, P = 0.073). However, rectal compliance was not changed much after nCRT (1.4 ± 0.8 mL/mmHg vs 1.3 ± 0.7 mL/mmHg, P = 0.638). Haboubi et al., [18] in their histologic study after radiotherapy, demonstrated that acute changes of the rectum included mucosal edema and patchy fibroblastic proliferation in the lamina propria. They found that cellular epithelial damage appeared early after radiotherapy whereas architectural epithelial damage appeared late after radiotherapy. This may explain the unchanged rectal compliance in early period after nCRT. The decrease of maximal RST and the maintenance of rectal compliance may imply that rectal hypersensitivity without the change of rectal elasticity was induced by nCRT in early period after nCRT.

Secondly, we intended to investigate the change of anal sphincter function after nCRT by comparing two groups divided by whether anal sphincter was irradiated or not by additional booster on peritumoral region. In this study, we compared changes of manometric data between before and after nCRT according to tumor location with 5 cm criteria. For patients with tumor below 5 cm from the anal verge, the field of additional booster on peritumoral region included the anal canal. However, in tumor above 5 cm from the anal verge, the anal canal and perineum were excluded from the field of additional booster. As shown in Table 3, MRP, MSP, sphincter asymmetry, and HPZ length, which reflect sphincter function, were not significantly different between these two groups. This means that irradiation on sphincter complex did not affect sphincter function in short-term period after nCRT. This is similar to other studies that compared changes of manometric data between mid and low rectal cancer after nCRT [11,19].

Finally, we investigated the change of anorectal function according to tumor response. For this, we had observed the changes of manometric data according to the tumor response for nCRT, classifying four factors; change of tumor gross morphology, CIR, T stage, and tumor volume. In this study, rectal compliance (P = 0.035) and the maximal RST (P = 0.086) were lower in patients who had ulcerofungating type than in ulceroinfiltrative type before nCRT.

This outcome can be interpreted as the fungating component of tumor might affect anorectal function negatively, which led to bad influence on defecation symptom of those patients before nCRT. However, we observed that most patients (22/30, 73.3%) showed changes of tumor gross morphology from ulcerofungating type to ulceroinfiltrative type after nCRT. As shown in Table 3, the change of tumor gross morphology between before and after nCRT did not affect to the change of anorectal function. This finding shows that the response of fungating component of tumor to nCRT doesn’t significantly relate to the change of anorectal function. Lee et al. [20] demonstrated that preoperative rectal reservoir function is altered by the existence of a tumor and tumor infiltration may alter the anorectal function because the ulceroinfiltrative type was correlated with intramural distal spread. Thus, it can be said that we should focus on infiltrating components rather than fungating components when we evaluate rectal reservoir and anorectal function. In this study, tumor volume, CIR, and T stage were examined to evaluate the response of infiltrating component to nCRT. Though the fungating components take a significant sum in tumor volume, we assumed that infiltrating components should be reduced in some degrees if there was a volume reduction at all. Considering the volume reduction after nCRT, we could observe that the RST for desire to defecate was improved in the patients who had tumor which was decreased more than 70% of its volume. From these findings, we thought that marked volume reduction including infiltrative component of tumor could improve anorectal function after nCRT. We observed that as the CIR was higher, the maximal RST (P = 0.086) and the rectal compliance (P = 0.017) were lower before nCRT. This may explain that the extent of infiltrative component of tumor can be involved in the anorectal function before nCRT. After nCRT, although the changes of the maximal RST and rectal compliance were not significantly different between patients with and without reduced CIR, the RST for desire to defecate in patients with reduced CIR was marginally higher than that in patients without reduced CIR (P = 0.099). This finding may suggest that in addition to the effect of radiation on normal tissue, the effect of radiation on tumor can affect the rectal sensory as well, to improve anorectal function, inversely. Although there was no significant difference in RST according to T stage before nCRT, the RST for desire to defecate was significantly higher in patients with down staging after nCRT than in patients without down staging (P = 0.016). However, these changes of manometric data resulted not from improving the RST for desire to defecate in patients with reduced CIR or T stage but from worsening in patients without those. This means that good tumor response with nCRT offset the negative effect for anorectal function, especially sensory function, worsened by nCRT.

Jang et al. [11] demonstrated that the observed decrease of maximal tolerance volume and rectal compliance was in contradiction with the improvement of clinical symptoms observed after nCRT. They explained the reason by a positive effect on clinical impact of tumor downsizing. Although these findings are similar to our study, our study suggested more concrete result for the relationship between the change of anorectal function and the tumor response. In summary, the nCRT might affect the RST positively when the infiltrative component of tumor had a response with booster dose for tumor, but irradiated rectum and sphincter complex might not be affected. Our results give the information about short-term anorectal function in regard to future strategies like omitting major surgery in patients with complete response after nCRT or applying those findings (especially in the group with tumors located < 5 cm from anal verge) to patients with anal cancer, who are usually treated with chemoradiation alone, although higher doses and different chemotherapy agents are used.

This study has some limitations such as use of retrospective analysis, a small number of patients, and omitting evaluation of clinical symptoms. However, there are not any reports searching about the short-term relationship with quantitative assessment between anorectal function and tumor response after nCRT for rectal cancer.

## Conclusions

In conclusion, the nCRT did not impair overall sphincter function before radical operation significantly. In this study, when tumor was shrunk or down-staged, the RST for desire to defecate was increased. Especially, those patients who showed down-staging by transrectal ultrasonography had significantly increased rectal sensory threshold for desire to defecate. This finding may suggest that nCRT may be helpful for their defecatory function in patients with radiosensitive rectal cancer as well as local disease control, at least in the short-term period of nCRT.

## Competing interests

The authors declare that they have no competing interests.

## Authors’ contributions

A study conception and design: K and C. Acquisition of data: K, HJK, SHK, L, and C. Analysis and interpretation of data: K, HJK, JGK, S, and C. Drafting of manuscript: K, HJK, JGK, SHK, S, L, and C. Critical revision: K, HJK, and C. Final approval: K, HJK, JGK, SHK, S, L, and C. All authors read and approved the final manuscript.
